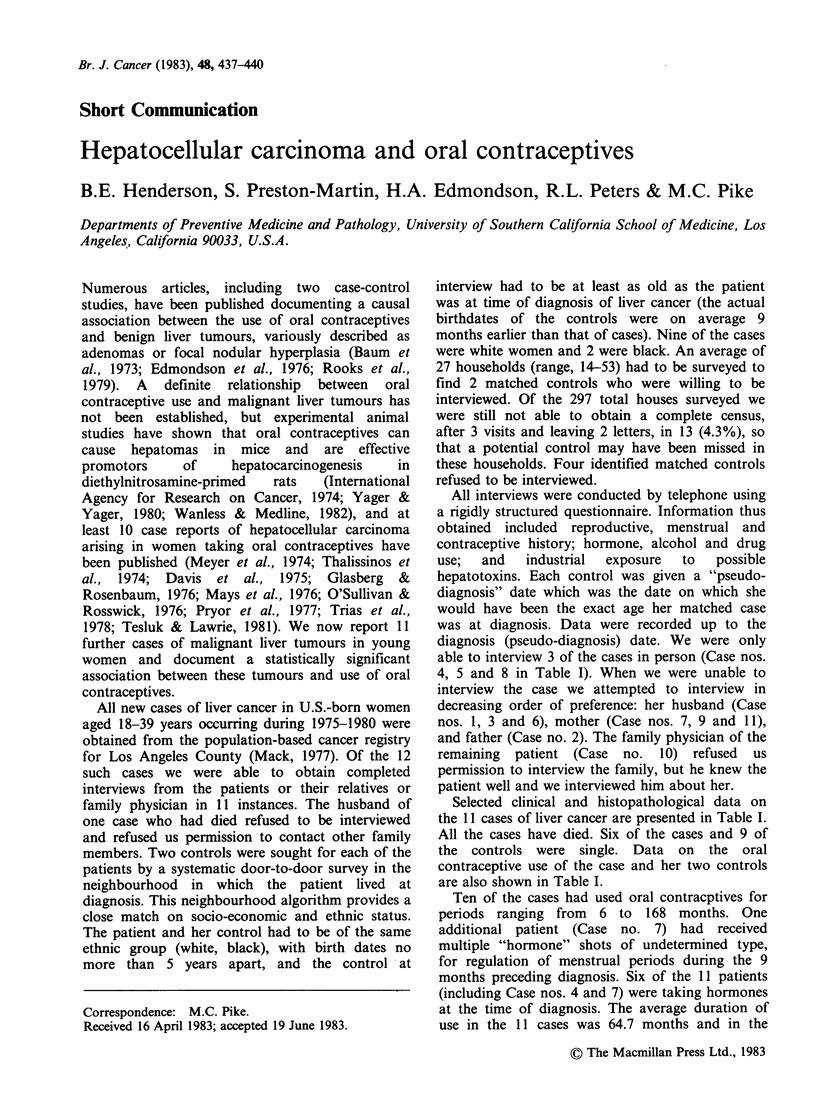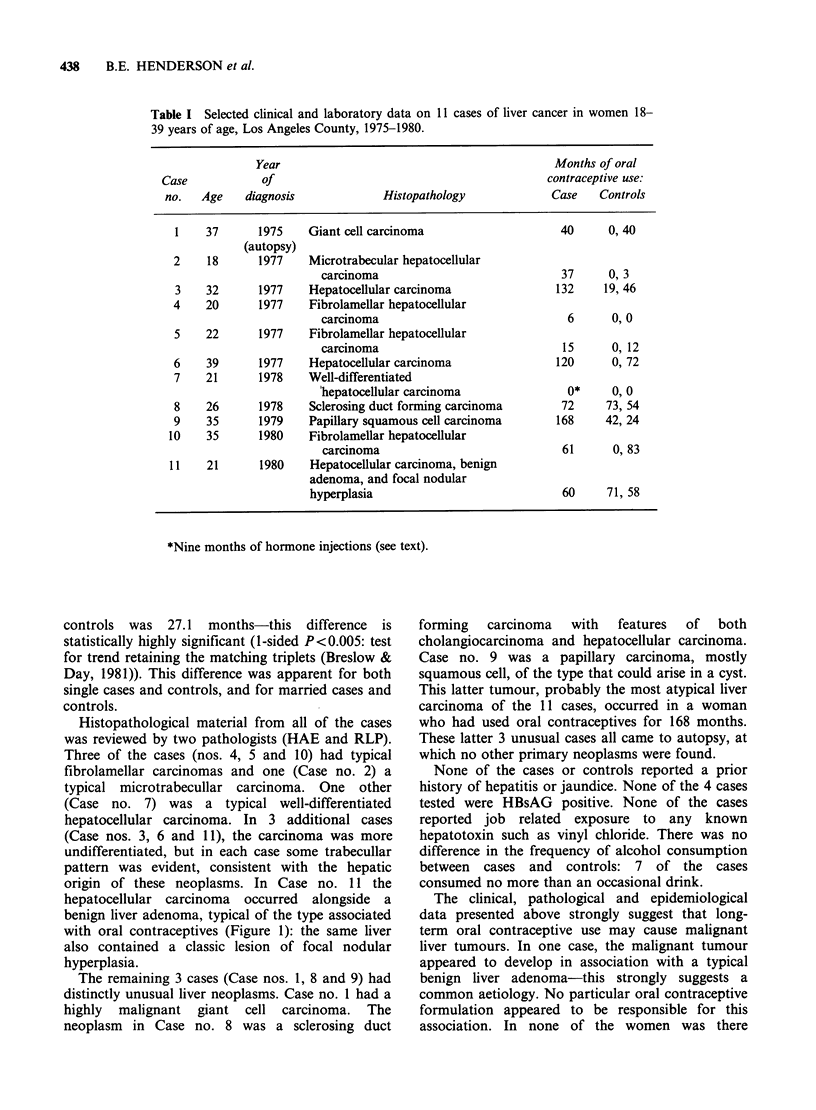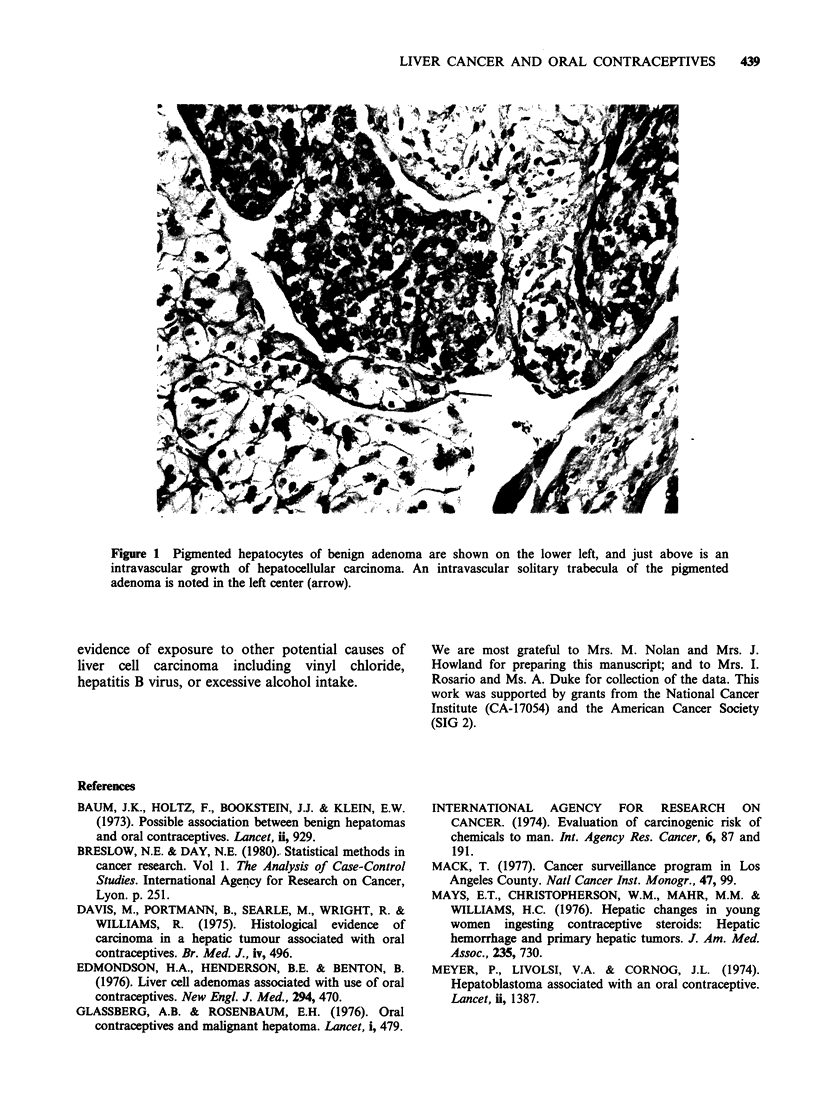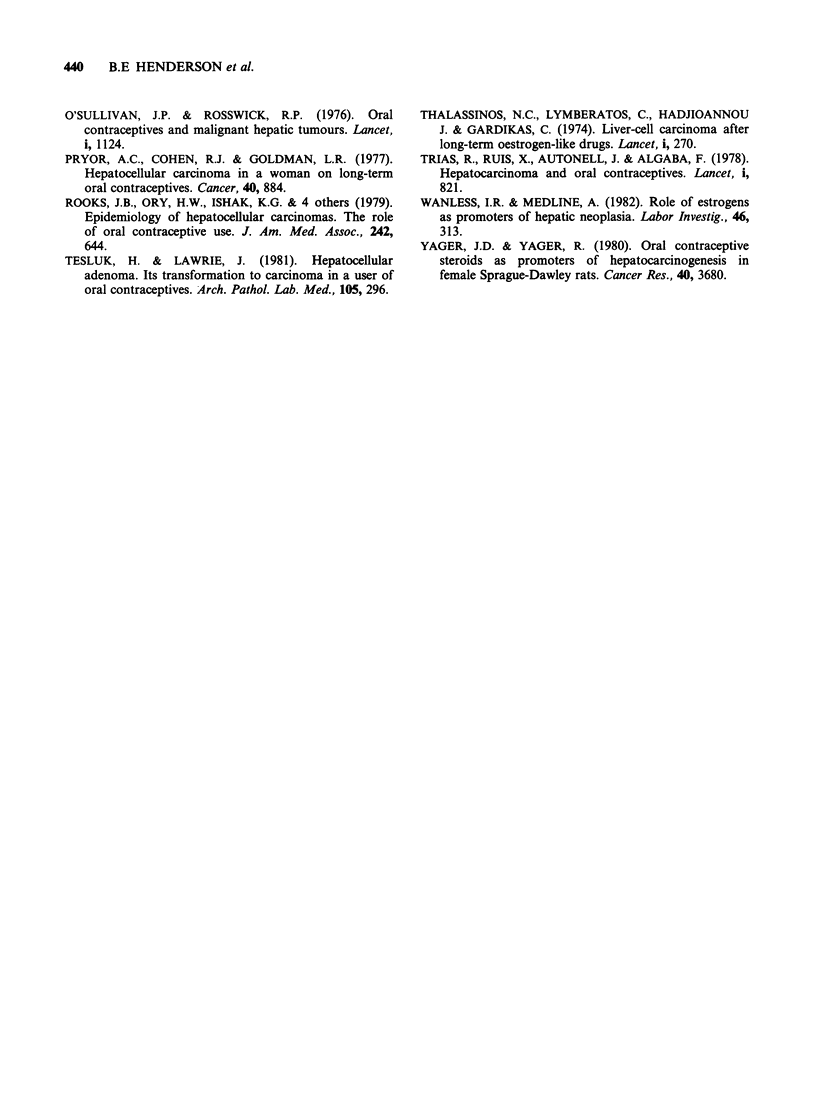# Hepatocellular carcinoma and oral contraceptives.

**DOI:** 10.1038/bjc.1983.210

**Published:** 1983-09

**Authors:** B. E. Henderson, S. Preston-Martin, H. A. Edmondson, R. L. Peters, M. C. Pike

## Abstract

**Images:**


*Br. J. Cancer* (1983), **48**, 437–440

**Short Communication**

### Hepatocellular carcinoma and oral contraceptives

B.E. Henderson, S. Preston-Martin, H.A. Edmondson, R.L. Peters & M.C. Pike

*Departments of Preventive Medicine and Pathology, University of Southern California School of Medicine, Los Angeles, California 90033, U.S.A.*

Numerous articles, including two case-control studies, have been published documenting a causal association between the use of oral contraceptives and benign liver tumours, variously described as adenomas or focal nodular hyperplasia (Baum *et al.*, 1973; Edmondson *et al.*, 1976; Rooks *et al.*, 1979). A definite relationship between oral contraceptive use and malignant liver tumours has not been established, but experimental animal studies have shown that oral contraceptives can cause hepatomas in mice and are effective promotors of hepatocarcinogenesis in diethylnitrosamine-primed rats (International Agency for Research on Cancer, 1974; Yager & Yager, 1980; Wanless & Medline, 1982), and at least 10 case reports of hepatocellular carcinoma arising in women taking oral contraceptives have been published (Meyer *et al.*, 1974; Thalissinos *et al.*, 1974; Davis *et al.*, 1975; Glasberg & Rosenbaum, 1976; Mays *et al.*, 1976; O'Sullivan & Rosswick, 1976; Pryor *et al.*, 1977; Trias *et al.*, 1978; Tesluk & Lawrie, 1981). We now report 11 further cases of malignant liver tumours in young women and document a statistically significant association between these tumours and use of oral contraceptives.

All new cases of liver cancer in U.S.-born women aged 18–39 years occurring during 1975–1980 were obtained from the population-based cancer registry for Los Angeles County (Mack, 1977). Of the 12 such cases we were able to obtain completed interviews from the patients or their relatives or family physician in 11 instances. The husband of one case who had died refused to be interviewed and refused us permission to contact other family members. Two controls were sought for each of the patients by a systematic door-to-door survey in the neighbourhood in which the patient lived at diagnosis. This neighbourhood algorithm provides a close match on socio-economic and ethnic status. The patient and her control had to be of the same ethnic group (white, black), with birth dates no more than 5 years apart, and the control at interview had to be at least as old as the patient was at time of diagnosis of liver cancer (the actual birthdates of the controls were on average 9 months earlier than that of cases). Nine of the cases were white women and 2 were black. An average of 27 households (range, 14–53) had to be surveyed to find 2 matched controls who were willing to be interviewed. Of the 297 total houses surveyed we were still not able to obtain a complete census, after 3 visits and leaving 2 letters, in 13 (4.3%), so that a potential control may have been missed in these households. Four identified matched controls refused to be interviewed.

All interviews were conducted by telephone using a rigidly structured questionnaire. Information thus obtained included reproductive, menstrual and contraceptive history; hormone, alcohol and drug use; and industrial exposure to possible hepatotoxins. Each control was given a "pseudo-diagnosis" date which was the date on which she would have been the exact age her matched case was at diagnosis. Data were recorded up to the diagnosis (pseudo-diagnosis) date. We were only able to interview 3 of the cases in person (Case nos. 4, 5 and 8 in Table I). When we were unable to interview the case we attempted to interview in decreasing order of preference: her husband (Case nos. 1, 3 and 6), mother (Case nos. 7, 9 and 11), and father (Case no. 2). The family physician of the remaining patient (Case no. 10) refused us permission to interview the family, but he knew the patient well and we interviewed him about her.

Selected clinical and histopathological data on the 11 cases of liver cancer are presented in Table I. All the cases have died. Six of the cases and 9 of the controls were single. Data on the oral contraceptive use of the case and her two controls are also shown in Table I.

Ten of the cases had used oral contracptives for periods ranging from 6 to 168 months. One additional patient (Case no. 7) had received multiple "hormone" shots of undetermined type, for regulation of menstrual periods during the 9 months preceding diagnosis. Six of the 11 patients (including Case nos. 4 and 7) were taking hormones at the time of diagnosis. The average duration of use in the 11 cases was 64.7 months and in the

Correspondence: M.C. Pike.
Received 16 April 1983; accepted 19 June 1983.

© The Macmillan Press Ltd., 1983

**Table I** Selected clinical and laboratory data on 11 cases of liver cancer in women 18–39 years of age, Los Angeles County, 1975–1980.

| *Case no.* | *Age* | *Year of diagnosis* | *Histopathology* | *Months of oral contraceptive use: Case* | *Controls* |
|---|---|---|---|---|---|
| 1 | 37 | 1975 (autopsy) | Giant cell carcinoma | 40 | 0, 40 |
| 2 | 18 | 1977 | Microtrabecular hepatocellular carcinoma | 37 | 0, 3 |
| 3 | 32 | 1977 | Hepatocellular carcinoma | 132 | 19, 46 |
| 4 | 20 | 1977 | Fibrolamellar hepatocellular carcinoma | 6 | 0, 0 |
| 5 | 22 | 1977 | Fibrolamellar hepatocellular carcinoma | 15 | 0, 12 |
| 6 | 39 | 1977 | Hepatocellular carcinoma | 120 | 0, 72 |
| 7 | 21 | 1978 | Well-differentiated hepatocellular carcinoma | 0* | 0, 0 |
| 8 | 26 | 1978 | Sclerosing duct forming carcinoma | 72 | 73, 54 |
| 9 | 35 | 1979 | Papillary squamous cell carcinoma | 168 | 42, 24 |
| 10 | 35 | 1980 | Fibrolamellar hepatocellular carcinoma | 61 | 0, 83 |
| 11 | 21 | 1980 | Hepatocellular carcinoma, benign adenoma, and focal nodular hyperplasia | 60 | 71, 58 |

*Nine months of hormone injections (see text).

controls was 27.1 months—this difference is statistically highly significant (1-sided $P<0.005$: test for trend retaining the matching triplets (Breslow & Day, 1981)). This difference was apparent for both single cases and controls, and for married cases and controls.

Histopathological material from all of the cases was reviewed by two pathologists (HAE and RLP). Three of the cases (nos. 4, 5 and 10) had typical fibrolamellar carcinomas and one (Case no. 2) a typical microtrabecullar carcinoma. One other (Case no. 7) was a typical well-differentiated hepatocellular carcinoma. In 3 additional cases (Case nos. 3, 6 and 11), the carcinoma was more undifferentiated, but in each case some trabecullar pattern was evident, consistent with the hepatic origin of these neoplasms. In Case no. 11 the hepatocellular carcinoma occurred alongside a benign liver adenoma, typical of the type associated with oral contraceptives (Figure 1): the same liver also contained a classic lesion of focal nodular hyperplasia.

The remaining 3 cases (Case nos. 1, 8 and 9) had distinctly unusual liver neoplasms. Case no. 1 had a highly malignant giant cell carcinoma. The neoplasm in Case no. 8 was a sclerosing duct forming carcinoma with features of both cholangiocarcinoma and hepatocellular carcinoma. Case no. 9 was a papillary carcinoma, mostly squamous cell, of the type that could arise in a cyst. This latter tumour, probably the most atypical liver carcinoma of the 11 cases, occurred in a woman who had used oral contraceptives for 168 months. These latter 3 unusual cases all came to autopsy, at which no other primary neoplasms were found.

None of the cases or controls reported a prior history of hepatitis or jaundice. None of the 4 cases tested were HBsAG positive. None of the cases reported job related exposure to any known hepatotoxin such as vinyl chloride. There was no difference in the frequency of alcohol consumption between cases and controls: 7 of the cases consumed no more than an occasional drink.

The clinical, pathological and epidemiological data presented above strongly suggest that long-term oral contraceptive use may cause malignant liver tumours. In one case, the malignant tumour appeared to develop in association with a typical benign liver adenoma—this strongly suggests a common aetiology. No particular oral contraceptive formulation appeared to be responsible for this association. In none of the women was there

**Figure 1** Pigmented hepatocytes of benign adenoma are shown on the lower left, and just above is an intravascular growth of hepatocellular carcinoma. An intravascular solitary trabecula of the pigmented adenoma is noted in the left center (arrow).

evidence of exposure to other potential causes of liver cell carcinoma including vinyl chloride, hepatitis B virus, or excessive alcohol intake.

We are most grateful to Mrs. M. Nolan and Mrs. J. Howland for preparing this manuscript; and to Mrs. I. Rosario and Ms. A. Duke for collection of the data. This work was supported by grants from the National Cancer Institute (CA-17054) and the American Cancer Society (SIG 2).

### References

BAUM, J.K., HOLTZ, F., BOOKSTEIN, J.J. & KLEIN, E.W. (1973). Possible association between benign hepatomas and oral contraceptives. *Lancet*, **ii**, 929.

BRESLOW, N.E. & DAY, N.E. (1980). Statistical methods in cancer research. Vol 1. *The Analysis of Case-Control Studies*. International Agency for Research on Cancer, Lyon. p. 251.

DAVIS, M., PORTMANN, B., SEARLE, M., WRIGHT, R. & WILLIAMS, R. (1975). Histological evidence of carcinoma in a hepatic tumour associated with oral contraceptives. *Br. Med. J.*, **iv**, 496.

EDMONDSON, H.A., HENDERSON, B.E. & BENTON, B. (1976). Liver cell adenomas associated with use of oral contraceptives. *New Engl. J. Med.*, **294**, 470.

GLASSBERG, A.B. & ROSENBAUM, E.H. (1976). Oral contraceptives and malignant hepatoma. *Lancet*, **i**, 479.

INTERNATIONAL AGENCY FOR RESEARCH ON CANCER. (1974). Evaluation of carcinogenic risk of chemicals to man. *Int. Agency Res. Cancer*, **6**, 87 and 191.

MACK, T. (1977). Cancer surveillance program in Los Angeles County. *Natl Cancer Inst. Monogr.*, **47**, 99.

MAYS, E.T., CHRISTOPHERSON, W.M., MAHR, M.M. & WILLIAMS, H.C. (1976). Hepatic changes in young women ingesting contraceptive steroids: Hepatic hemorrhage and primary hepatic tumors. *J. Am. Med. Assoc.*, **235**, 730.

MEYER, P., LIVOLSI, V.A. & CORNOG, J.L. (1974). Hepatoblastoma associated with an oral contraceptive. *Lancet*, **ii**, 1387.

O'SULLIVAN, J.P. & ROSSWICK, R.P. (1976). Oral contraceptives and malignant hepatic tumours. *Lancet*, **i**, 1124.

PRYOR, A.C., COHEN, R.J. & GOLDMAN, L.R. (1977). Hepatocellular carcinoma in a woman on long-term oral contraceptives. *Cancer*, **40**, 884.

ROOKS, J.B., ORY, H.W., ISHAK, K.G. & 4 others (1979). Epidemiology of hepatocellular carcinomas. The role of oral contraceptive use. *J. Am. Med. Assoc.*, **242**, 644.

TESLUK, H. & LAWRIE, J. (1981). Hepatocellular adenoma. Its transformation to carcinoma in a user of oral contraceptives. *Arch. Pathol. Lab. Med.*, **105**, 296.

THALASSINOS, N.C., LYMBERATOS, C., HADJIOANNOU J. & GARDIKAS, C. (1974). Liver-cell carcinoma after long-term oestrogen-like drugs. *Lancet*, **i**, 270.

TRIAS, R., RUIS, X., AUTONELL, J. & ALGABA, F. (1978). Hepatocarcinoma and oral contraceptives. *Lancet*, **i**, 821.

WANLESS, I.R. & MEDLINE, A. (1982). Role of estrogens as promoters of hepatic neoplasia. *Labor Investig.*, **46**, 313.

YAGER, J.D. & YAGER, R. (1980). Oral contraceptive steroids as promoters of hepatocarcinogenesis in female Sprague-Dawley rats. *Cancer Res.*, **40**, 3680.